# Non-Destructive Evaluation of HTV’s Thermal-Oxidative Aging Using Terahertz Dielectric Spectroscopy

**DOI:** 10.3390/ma18174176

**Published:** 2025-09-05

**Authors:** Tengyi Zhang, Li Cheng, Shuo Zhang, Bo Tao, Yipu Tang

**Affiliations:** 1 School of Electrical Engineering, Chongqing University, Chongqing 400044, China; 2 State Grid Sichuan Electric Power Company Luzhou Power Supply Company, Luzhou 646000, China

**Keywords:** HTV, thermal oxidation aging, terahertz dielectric spectrum, infrared spectrum, Double Debye model, insulator life prediction

## Abstract

Thermal oxidative aging failure of high-temperature vulcanized silicone rubber (HTV) in high-voltage insulators is the core hidden danger of power grid security. In this study, terahertz time domain spectroscopy (THz-TDS) and attenuated total reflection infrared spectroscopy (ATR-FTIR) were combined to reveal the quantitative structure–activity relationship between dielectric response and chemical group evolution of HTV during accelerated aging at 200 °C for 80 days. In this study, HTV flat samples were made in the laboratory, and the dielectric spectrum of HTV in the range of 0.1 THz to 0.4 THz was extracted by a terahertz time–domain spectrum platform. ATR-FTIR was used to analyze the functional group change trend of HTV during aging, and the three-stage evolution of the dielectric real part (0.16 THz), the dynamics of the carbonyl group, the monotonic rise of the dielectric imaginary part (0.17 THz), and the linear response of silicon-oxygen bond breaking were obtained by combining the double Debye relaxation theory. Finally, three aging stages of HTV were characterized by dielectric loss angle data. The model can warn about the critical point of early oxidation and main chain fracture and identify the risk of insulation failure in advance compared with traditional methods. This study provides a multi-scale physical basis for nondestructive life assessment in a silicon rubber insulator.

## 1. Introduction

High-temperature vulcanized silicone rubber (HTV) has become the core material of composite insulators for high-voltage transmission lines because of its excellent hydrophobicity, ultraviolet aging resistance, and electrical insulation stability in extreme environments. Compared with traditional ceramic or glass insulators, its unique surface characteristics can effectively suppress leakage current and significantly reduce the risk of pollution flashover accidents [1]. However, under the synergistic effect of long-term thermal-oxygen stress, the molecular chain of HTV will deteriorate irreversibly: fracture of the main chain will lead to a decrease in mechanical strength, embrittlement delamination will lead to fracture of the umbrella skirt, and oxidation of side groups will lead to the increase of dielectric loss [2]. At the same time, the hydrophobicity and hydrophobic mobility of HTV will be permanently reduced, greatly increasing the probability of pollution flashover. If this aging process is not monitored in time, it will lead to insulator failure and cause economic losses. Therefore, it is of great engineering significance to accurately diagnose the aging state of HTV for preventing power grid flashover accidents and optimizing the insulator replacement strategy. At present, it is urgent to establish the correlation mechanism between the micro-deterioration of materials and macro-electrical properties and to provide a theoretical basis for insulator residual life evaluation [3,4].

At present, the evaluation of the aging state of silicone rubber is mainly to measure its electrical, chemical, and mechanical characteristics [5]. Lin, Y. [6] and others characterized the cross-linking density of LSR by measuring the dielectric loss tangent, relative dielectric constant, and volume resistivity, and Li, G.C. [7] pointed out that the relationship between the change in chemical groups and the macroscopic electrical properties needs the help of empirical models and is sensitive to the sample surface. The detection depth of FTIR is limited (1–5 μm), and XPS needs a vacuum environment and cannot be implemented in every situation [8]. The mechanical property testing method is characterized by tensile strength, hardness tester, etc. [9,10]. This method needs destructive sampling, and the material has entered the late aging stage when the elongation at break drops to 50%, so it is impossible to realize an early warning. Moreover, off-line detection will lead to a monitoring blind spot in the running state of insulators, and there is a lack of a fast nondestructive testing method on site at present.

The terahertz frequency band (0.1–0.4 THz) directly corresponds to the movement of silicone rubber molecular segments (Si-O bond torsional vibration: 0.25 THz) and the relaxation process of polar groups, which makes the dielectric response intrinsically related to the change in chemical structure. Its penetration depth is 3–5 mm, which is 1000 times higher than FTIR [11,12]. Non-ionizing radiation characteristics ensure zero damage to materials, and optical fiber coupling probes support live detection of equipment. At the same time, the dielectric constant of the sample is a complex number, which contains both polarization ability and loss mechanism information. The dual characteristic quantity overcomes the one-sidedness of a single chemical/electrical parameter [13].

In this study, the new HTV flat samples were aged for 80 days by a hot oxygen aging box. Then, the Shore hardness test and FTIR absorption spectrum analysis were carried out to verify the aging mechanism of HTV and the changes in functional groups in PDMS during aging. Then, the dielectric spectrum data of the samples were extracted by terahertz time–domain spectroscopy technology, the reasons for the changes in dielectric real and imaginary parts of HTV during aging were studied theoretically, and the aging law of HTV was analyzed. Finally, the aging state of HTV was fitted by using tanδ as the aging characteristic quantity, and the initial, middle, and final stages of HTV aging were characterized.

## 2. Materials and Methods

### 2.1. Materials

In this study, HTV for composite insulators was selected. The main raw materials of HTV were a rubber base (polydimethylsiloxane (PDMS)) and filler. The filler mainly included flame-retardant aluminum trihydrate (ATH) and a reinforcing agent (SiO_2_), as shown in Table 1, and there were a few additives, including hydroxyl silicone oil, colorant, and a vulcanizer. The content of additives was generally about 10% *w*/*w*. The manufacturing method of HTV was to mix PDMS with a filler. In this study, PDMS content was 36% *w*/*w*, ATH content was 45% *w*/*w*, silica content was 15% *w*/*w*, and additive (including hydroxyl silicone oil, colorant, and vulcanizer) was 4% *w*/*w*. PDMS and the filler were fully mixed in a mixer and heat-treated at 150 °C for 2 h, then the vulcanizing agent was added, and vulcanization molding was carried out in a flat vulcanizer. The thickness was controlled at 2 mm ± 0.1 mm.

Then, the finished HTV flat sample was put into a thermal oxygen aging box for aging treatment. Because the decomposition temperature of ATH is 220 °C, in order to avoid premature decomposition of the filler caused by too high of a temperature, the aging temperature was set at 200 °C for 80 days.

### 2.2. Terahertz Time Domain Spectrum Detection Platform

#### 2.2.1. Terahertz Detection Platform

**Figure 1:**
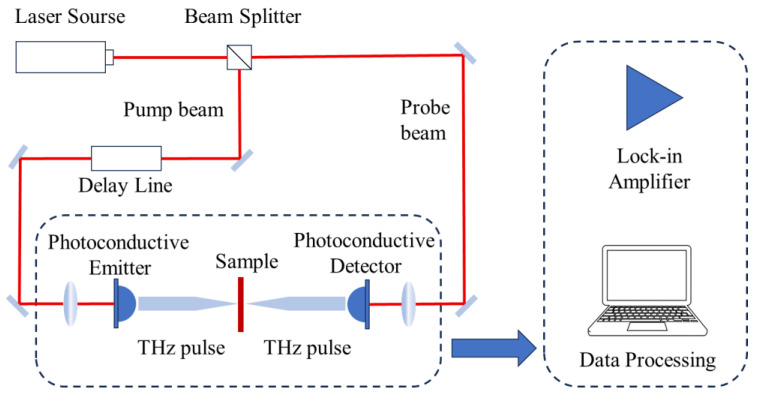
Terahertz detection platform.

In this study, Teraview TeraPulse Lx (Cambridge, UK) was used as the terahertz detection platform. The laser type of this instrument conforms to IEC 60825-1 1 standard [14]. The platform is mainly composed of terahertz signal transmitting module, detection module, and signal processing module, as shown in Figure 1. A femtosecond laser emits a beam of laser light to a beam splitter to generate pump light and probe light, and the pump light reaches a terahertz generator after passing through a delay device to generate terahertz pulse signals. The terahertz pulse passes through the sample to be tested and reaches the terahertz detector at the same time as the detection light; finally, it reaches the computer for analysis and processing after passing through the lock-in amplifier.

#### 2.2.2. THz Dielectric Spectrum Extraction Methods

The time–domain electric field signal $E_{sam}\left(t\right)$ of the terahertz wave transmission sample and the reference signal $E_{ref}\left(t\right)$ of the non-transmission sample were obtained by the terahertz time–domain spectrum the detection platform, and the frequency–domain signals $E_{sam}\left(\omega \right)$ and $E_{ref}\left(\omega \right)$ was obtained by Fourier transform.

The transmission signal is the ratio of the sample to the reference signal, as shown in Formula (1).

(1) $$E_{0}\left(\omega \right)=\frac{E_{sam}\left(\omega \right)}{E_{ref}\left(\omega \right)}=\left|T\left(\omega \right)\right|e^{j\Delta \phi \left(\omega \right)}$$

where $\left|T\left(\omega \right)\right|$ is the amplitude attenuation ratio and $\Delta \phi \left(\omega \right)$ is the phase delay difference.

In order to obtain the terahertz complex dielectric constant of the sample, it is necessary to calculate the complex refractive index $\tilde{n}=n-j\kappa$, as shown in Formula (2).

(2) $$T\left(\omega \right)=\frac{4\tilde{n}}{{\left(\tilde{n}+1\right)}^{2}}\cdot exp\left[-j\frac{\omega }{c}\left(\tilde{n}-1\right)d\right]$$

where d is the thickness of the sample, c is the speed of light in vacuum, and ω is the angular frequency. The amplitude and phase equations are separated by the transmission coefficient.

(3) $$\left|T\left(\omega \right)\right|=\frac{4\sqrt{n^{2}+\kappa^{2}}}{{\left(n+1\right)}^{2}+\kappa^{2}}exp\left(-\frac{\omega \kappa d}{c}\right)$$

(4) $$\Delta \phi \left(\omega \right)=\frac{\omega d}{c}\left(n-1\right)+\arg\left[\frac{4\tilde{n}}{{\left(\tilde{n}+1\right)}^{2}}\right]$$

Silicone rubber has weak absorption $\left(\kappa \ll n\right)$ for terahertz waves, and Formulas (5) and (6) can be approximately simplified as follows:

(5) $$n\left(\omega \right)\approx 1+\frac{c\Delta \phi \left(\omega \right)}{\omega d}$$

(6) $$\kappa \left(\omega \right)\approx \frac{c}{\omega d}\ln\left[\frac{4n\left(\omega \right)}{\left|T\left(\omega \right)\right|{\left(n\left(\omega \right)+1\right)}^{2}}\right]$$

The relationship between the dielectric constant $\tilde{\varepsilon }=\varepsilon^{\prime }-j\varepsilon^{\prime \prime }$ and refractive index $\tilde{n}$ is $\tilde{\varepsilon }={\tilde{n}}^{2}$, and the values of $\varepsilon^{\prime }$ and $\varepsilon^{\prime \prime }$ can be obtained.

(7) $$\left\{\begin{array}{c} \varepsilon^{\prime }=n^{2}-\kappa^{2}\\ \varepsilon^{\prime \prime }=2n\kappa \end{array}\right.$$

### 2.3. Chemical Property Detection

The FTIR spectrometer Nicolet iS20 (Thermo Fisher Scientific, Waltham, MA, USA) was used to analyze the functional groups of aging HTV samples. The signal-to-noise ratio of the equipment is 50,000:1, the standard spectral resolution is 4 cm^−1^, and the wave number accuracy is 0.0008 cm^−1^. Because the group changes in the aging process of HTV have absorption peaks from 700 to 3500 cm^−1^, the wavenumbers test range of this study is 400–4000 cm^−1^. The ATR crystal was diamond, the detector was a deuterated triglycine sulfate (DTGS) detector, and the beamsplitter was a germanium-coated potassium bromide (KBr) beamsplitter. Because the aging flat sample of HTV needs to be kept in the original state during detection, the ATR-FTIR measurement method is more sensitive to changes in the oxidation surface groups of HTV in the early stage.

## 3. Results and Discussion

### 3.1. THz Dielectric Spectrum

Silicone rubber is a dielectric material. When the sample is in an alternating electric field, the molecules in silicone rubber will be polarized. Polarization types range from low frequency to high frequency, including electron polarization, atom polarization, orientation polarization, interface polarization, and vibration and rotation of molecular energy level. In this study, the relaxation polarization response of molecules is considered because the frequency domain of an alternating electric field is in the terahertz frequency band. In HTV, the directional polarization and ionic polarization of the dipole mainly occur in the terahertz frequency range, and the polarization mode depends on different polar groups and the environmental temperature. At the same time, PDMS is a polymer, which has a low absorption of terahertz radiation, and the polar groups in the molecular chain greatly determine the polarization mode.

**Figure 2:**
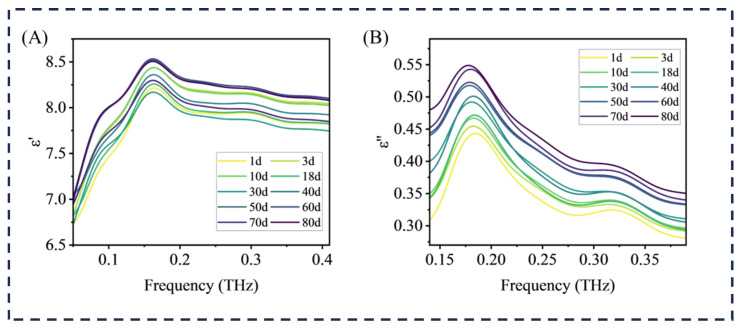
Dielectric constant of aging HTV sample: (**A**) real part of complex dielectric constant (**B**) imaginary part of complex dielectric constant.

The terahertz dielectric spectrum can show the change in molecular structure and polarization characteristics of HTV and calculate the terahertz dielectric spectrum of aging samples to analyze the polarity change in HTV during aging. As shown in Figure 2A,B, in the low frequency range (0–0.2 THz), the THz dielectric real part and imaginary part waveform patterns of the samples in each aging stage are consistent, with the peak value of the real part of the sample being around 0.161 THz and the peak value of the imaginary part being around 0.174. After exceeding 0.17 THz, both ε′ and ε″ show a downward trend, which is determined by the molecular dynamics response and the intrinsic characteristics of the material, and this trend is more obvious after thermal oxidation aging.

When the frequency of the alternating electric field is low, the dipole polarization process in dielectric materials can often keep pace with the change in the electric field, and the energy loss is small at this time, which is mainly caused by the conductive characteristics of the materials. In contrast, when the frequency of the external alternating electric field increases, the polarization motion of the dipole needs to break through the friction obstacle inside the material, and its changing rhythm will lag behind the change in the electric field. In this state, the relaxation polarization will become more and more significant because it takes extra energy to overcome the viscous resistance inside the material. Due to the change in PDMS structure in HTV during aging, it is necessary to further discuss the downward trend of the terahertz dielectric spectrum.

### 3.2. HTV Infrared Spectrum Analysis

**Figure 3:**
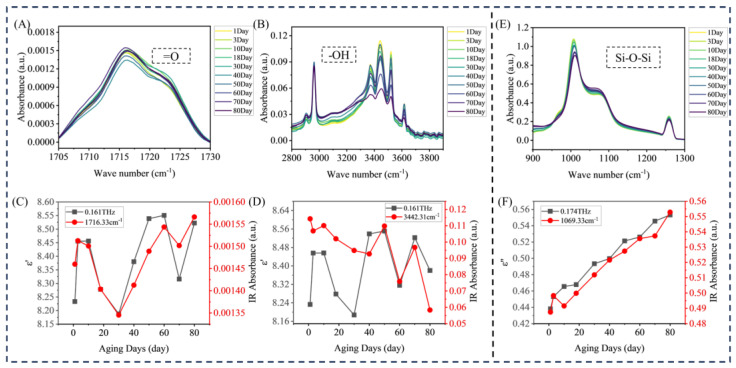
Relationship between functional group changes of HTV and terahertz dielectric spectrum: (**A**) carbonyl change, (**B**) hydroxyl change, (**C**) relationship between dielectric real part and carbonyl, (**D**) relationship between dielectric real part and hydroxyl, (**E**) Si-O-Si change, (**F**) relationship between dielectric imaginary part and Si-O-Si.

Under the condition of thermal oxidation aging of HTV, with the increase in aging time, the groups in HTV will obviously change, as shown in Figure 3. The main components of HTV are PDMS, ATH, and SiO_2_. Among them, the hydroxyl stretching vibration of ATH and SiO_2_ is in the range of 2800–3700, the stretching vibration of Si-O-Si is 1000–1100, skeleton stretching vibration of Si-O and Al-O is 1050–1100, and the stretching vibration of Si-C and Al-OH is 600–800 [15].

There are three main types of group changes during the aging process of HTV. As shown in Figure 3A, the carbonyl content will change dynamically in the middle and late period before aging, which includes two processes: 1650 cm^−1^ comes from the side group oxidation of PDMS, generating R-CH=C=O, and 1720 cm^−1^ comes from the main chain break and accumulating R-COOH. During the initial 0–30 days of aging, it showed a rapid upward trend, and at this time, the side chain oxidation was dominant. In the middle of aging, the infrared absorption decreased due to the volatilization of small molecules in HTV and the overflow of oligomers. In late aging, the main chain of PDMS was broken, compensating for absorption, and gradually stabilized [16,17].

As shown in Figure 3B, the hydroxyl peak appears around 3400 cm^−1^, which is derived from the hydrolysis of Si-O-Si and the oxidation of free radicals.

As shown in Figure 3E,F, the antisymmetric stretching peak (1070 cm^−1^) of Si-O-Si indicates the fracture of the PDMS backbone.

The terahertz dielectric response of HTV is in strict correspondence with the above chemical changes. As shown in Figure 3C,D, the carbonyl group (C=O) is a strong polar group, and its change in content directly affects dipole moment density, which synchronously drives $\varepsilon^{\prime }$ change.

In this study, the infrared absorption spectrum of HTV is semi-quantitative, so the peak value of the absorption spectrum is selected as the characteristic quantity. From the infrared spectrum data, the change in carbonyl-related characteristic peaks is obviously in the range of 1650–1800 cm^−1^. With the increase in aging time, the carbonyl peak intensity in this area showed a trend of first rising and then falling, then rising and remaining stable. This change reflects the dynamic change in carbonyl content in HTV during aging. In the early stage of aging, due to aging factors such as thermal oxygen, the molecular chain of HTV undergoes oxidation and other reactions, which leads to the formation of the carbonyl group and the increase in its content, so the intensity of the corresponding peak is enhanced. With the further development of aging, some carbonyl groups may participate in other reactions or structural adjustment, which makes the content decrease and the peak intensity weaken. In the later stage of aging, new oxidation products or stable structures are gradually formed, the carbonyl content increases again and tends toward a stable state, and the peak intensity also changes. Similarly, the characteristic peaks of hydroxyl groups in the range of 3000–3600 cm^−1^ also show a similar trend. In the early stage of aging, the degradation of the HTV molecular chain and other reactions promoted the formation of hydroxyl groups, and the peak strength increased. In the middle stage, due to the rearrangement of the molecular chain structure and other factors, hydroxyl groups participated in the reaction, the content decreased, and the peak intensity decreased. In the later period, as the aging reaction continued, new hydroxyl-related structures were generated and stabilized, and the peak intensity rose again and stabilized.

Correspondingly, at a frequency of 0.16 THz, the numerical variation trend of the real part of the THz dielectric spectrum is highly consistent with the variation trend of carbonyl and hydroxyl in the above infrared spectrum, which also rises first and then falls, then rises and tends to be stable. This shows that the real part of the terahertz dielectric spectrum is extremely sensitive to the changes related to carbonyl and hydroxyl groups in the molecular structure of HTV at this frequency. However, at the frequency of 0.17 THz, the imaginary part of the THz dielectric spectrum shows a continuous upward trend, which is consistent with the changing trend of Si-O-Si content in the range of 900–1200 cm^−1^ in the infrared spectrum.

Pearson analysis was performed on terahertz dielectric data and infrared data of the sample, and the results are shown in Table 2.

It can be found that the carbonyl peak of 1716 cm^−1^ is significantly correlated with the dielectric real part of 0.161 THz, and the hydroxyl peak of 3442 cm^−1^ is not correlated with the dielectric real part in the early stage of aging, but it is significantly correlated after aging for more than 40 days. The absorption peak of 1069 cm^−1^ Si-O-Si is significantly correlated with the imaginary part of 0.174 THz.

### 3.3. Aging Mechanism of HTV

In order to further analyze the dielectric data of HTV samples during aging, the reasons for the change were explained by the movement difference at the molecular scale. The Debye relaxation model is a classical theory to describe the relaxation behavior of a polar dielectric in an alternating electric field. When polar materials (such as polymers) are in an electric field, the polar molecules inside them will be oriented along the direction of the electric field, which is called dielectric polarization. However, if the electric field is high-frequency AC, the molecular orientation movement lags behind the change in the electric field due to the interaction between molecules. When the direction of the electric field changes, the molecules cannot instantly turn around, which is called dielectric relaxation. Relaxation time τ is a key parameter, which represents the average time required for polar molecules to adjust from an equilibrium orientation state to a new state.

The terahertz dielectric spectrum of HTV samples was tested by experiments, and the Debye model was fitted. The Debye model describes the polarization relaxation behavior of the polar dielectric under an alternating electric field, and its complex dielectric constant was expressed as

(8) $$\tilde{\varepsilon }\left(\omega \right)=\varepsilon_{\infty }+\frac{\varepsilon_{s}-\varepsilon_{\infty }}{1+j\omega \tau }$$

(9) $$\varepsilon^{\prime }\left(\omega \right)=\varepsilon_{\infty }+\frac{\varepsilon_{s}-\varepsilon_{\infty }}{1+{\left(\omega \tau \right)}^{2}}$$

(10) $$\varepsilon^{\prime \prime }\left(\omega \right)=\frac{\left(\varepsilon_{s}-\varepsilon_{\infty }\right)\omega \tau }{1+{\left(\omega \tau \right)}^{2}}$$

where $\varepsilon_{s}$ is the lens dielectric constant; $\varepsilon_{\infty }$ is the optical frequency dielectric constant; τ is the characteristic relaxation time, indicating the molecular dipole steering rate; and ω is the angular frequency.

Because HTV has multiple relaxation processes in the range of 0.1–0.4 THz, and not only a single relaxation time τ, the Debye original model cannot distinguish the difference between and contribution of oxidation/chain breakage, and the fitting data has a big error, so this study uses the fast and slow Debye model to analyze HTV samples.

The fast and slow Debye model is shown in Formula (11).

(11) $$\tilde{\varepsilon }\left(\omega \right)=\varepsilon_{\infty }+\frac{\Delta \varepsilon_{\mathrm{fast}}}{1+j\omega \tau_{\mathrm{fast}}}+\frac{\Delta \varepsilon_{\mathrm{slow}}}{1+j\omega \tau_{\mathrm{slow}}}$$

where $\tau_{\mathrm{fast}}$ is the fast relaxation time, $\tau_{\mathrm{slow}}$ is the slow relaxation time, $\Delta \varepsilon_{\mathrm{fast}}$ is the fast relaxation dielectric constant, and $\Delta \varepsilon_{\mathrm{slow}}$ is the slow relaxation dielectric constant. For the fast relaxation process of HTV, the side group (-ch3, -oh) of PDMS rotates, and with the increase in oxidation products, τ will be prolonged. At the same time, the increase in polar small groups will increase the dielectric constant, which is positively related to the oxidation degree of HTV. For a slow relaxation time, the main chain of PDMS is broken, τ is shortened, and the dipole orientation polarization of the main chain is negatively correlated with the crosslinking density of PDMS.

The double Debye fitting parameters of HTV in the frequency band of 0.1–0.4 THz are shown in Table 3, which reflects the dual characteristics of molecular polarization relaxation and is closely related to the dynamic changes in carbonyl, hydroxyl, and Si-O-Si during aging.

The high-frequency limiting dielectric constant $\varepsilon_{\infty }$ originates from electron polarization and atom polarization and is significantly influenced by the molecular skeleton. The main chain composed of silicon-oxygen-silicon bonds (900–1200 cm^−1^) has a stable structure, high bond energy, and only weak vibration occurring in the terahertz frequency band, which makes $\varepsilon_{\infty }$ keep a low value and small fluctuation. $\tau_{\mathrm{fast}}$ corresponds to a high frequency response, and the intensity of $\Delta \varepsilon_{\mathrm{fast}}$ is related to the dipole rotation of hydroxyl (3000–3600 cm^−1^). The hydroxyl group is strong in polarity and small in size, can quickly respond to electric field changes under high-frequency excitation of 0.1–0.4 THz, and its concentration fluctuation affects polarization intensity through hydrogen bonding, which is consistent with the short-term fluctuation trend of terahertz real part. $\tau_{\mathrm{slow}}$ corresponds to low-frequency relaxation, and $\Delta \varepsilon_{\mathrm{slow}}$ mainly comes from the dipole orientation of carbonyl (1650–1800 cm^−1^). Carbonyl polar groups have strong interaction with surrounding molecules, so orientation in the electric field needs to overcome a greater resistance and longer relaxation time. The long-term change in its content changes the degree of polarization hysteresis through intermolecular interaction, which is closely related to the continuous upward trend of the THz imaginary part. The difference in Debye parameters is essentially the polarization dynamics difference between hydroxyl and carbonyl in the terahertz frequency band, and the rigid skeleton composed of Si-O-Si provides a stable molecular environment for the relaxation process.

### 3.4. Evaluation of HTV Aging Degree

#### 3.4.1. TGA of HTV Aging

**Figure 4:**
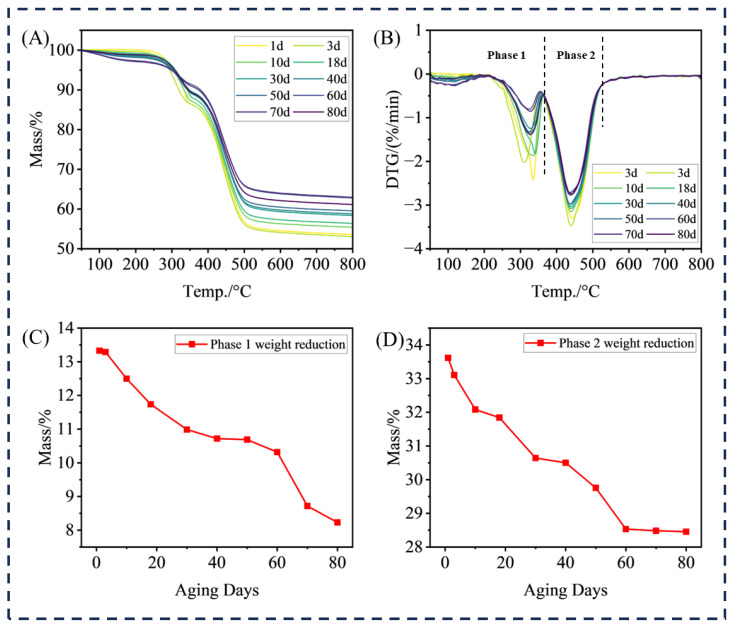
Thermogravimetric analysis of HTV (**A**) sample mass change, (**B**) sample mass change slope, (**C**) Phase 1 weightlessness weight, and (**D**) Phase 2 weightlessness weight.

The TGA data on HTV aging samples are shown in Figure 4.

The Figure 4A,B clearly show the two-stage characteristics of thermal decomposition of HTV samples. The first stage is the decomposition process of aluminum hydroxide (ATH), and the corresponding temperature range is 0–350 °C. In this stage, ATH is heated to remove crystal water. The second stage is the decomposition of the polydimethylsiloxane (PDMS) matrix, and the temperature range is concentrated at 350–550 °C. The main chain of PDMS is broken and volatilized. After thermal decomposition, the residual substances are mainly inorganic components such as silica (SiO_2_) and alumina, which are inert components with high-temperature resistance in HTV samples.

Further analysis of the influence of aging degree on thermal decomposition behavior shows that with the increase in the aging degree, the weight loss of HTV samples in the first stage (Figure 4C ATH decomposition) and the second stage (Figure 4D PDMS decomposition) tends to decrease, while the weight of the final residue increases accordingly. This phenomenon is mainly due to the change in the internal cross-linking structure of HTV during aging, which makes the thermal decomposition reaction between ATH and PDMS more difficult, and at the same time, the relative proportion of inert inorganic components is increased, which indirectly reflects the regulation of aging on the thermal stability of HTV.

#### 3.4.2. Aging State Model of HTV

In order to quantitatively evaluate the aging state of HTV, an Aging State Index (ASI), based on the tangent value of the dielectric loss angle (tanδ) was proposed in this study, and the aging process was divided into three stages: initial stage (0–35 days), middle stage (35–60 days), and final stage (60–80 days) combined with the accelerated aging experimental data of 0–80 days. tanδ is shown in Formula (12).

(12) $$\tan\delta =\frac{\varepsilon^{\prime \prime }}{\varepsilon^{\prime }}$$

**Figure 5:**
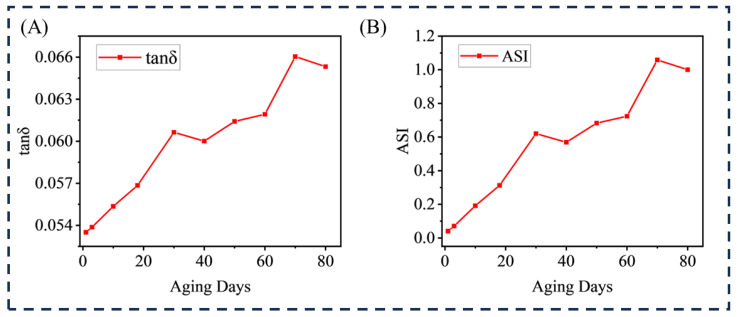
Evaluation of HTV aging: (**A**) relationship between HTV aging days and tan δ; (**B**) aging state model of HTV.

The tangent value of the dielectric loss angle has a high sensitivity and responds to the increase in polar groups and the decrease in polarization ability. In addition, tanδ is extremely sensitive to early oxidation at 0.17 THz. Therefore, the aging state model (ASI) can be constructed, as shown in Formula (13).

(13) $$ASI=\frac{\tan\delta \left(t\right)-\tan\delta_{0}}{\tan\delta_{max}-\tan\delta_{0}}$$

where $\tan\delta \left(t\right)$ is the measured value of dielectric loss angle at t days of aging, $\tan\delta_{0}$ is the reference value of unaged samples, and $\tan\delta_{max}$ is the maximum dielectric loss angle at the end of aging (80 days). ASI values range from 0 to 1, and the larger the value, the more serious the aging degree is. The aging evaluation is shown in Figure 5.

At the initial stage of aging, the molecular chain of HTV began to be slightly oxidized, and polar groups such as hydroxyl groups were initially formed, but the main chain structure of Si-O-Si remained intact, the aging index of HTV rose sharply, and $\tan\delta_{0.17}$ increased from 0.053 to 0.6. The rapid dipole relaxation of hydroxyl groups at this stage was the key reason for the sharp increase in the dielectric loss angle. In the middle stage of aging, with the intensification of the oxidation reaction, the carbonyl content increased significantly, some Si-O-Si broke, and the movement of cross-linked molecular segments was limited to some extent. tanδ was stable at about 0.066, and the slow relaxation of the carbonyl group and the loss caused by the change in Si-O-Si balanced each other, which made the overall dielectric loss angle have a relatively stable state, and the proportion of relaxation loss to conductivity loss was basically the same. At the end of aging, the molecular network structure was destroyed to a certain extent, the contents of the carbonyl group and hydroxyl group gradually stabilized, and micro-cracks began to appear in the material. At this stage, due to the increase in small molecular products produced by molecular chain degradation, the conductance loss becomes the main driving force for the rise in the dielectric loss angle, and the existence of microcracks will also aggravate the electric field distortion and further increase the energy loss. When the migration of small molecular products reaches equilibrium, the dielectric loss angle will stabilize, and the insulation performance of materials will enter the final stage of aging.

## 4. Conclusions

In this paper, a nondestructive testing method based on terahertz time domain spectroscopy technology is proposed. By analyzing the complex dielectric constant of HTV in the range of 0–0.2 THz, the aging degree of HTV under thermal oxidation aging can be judged, and the following conclusions are obtained.

In this study, the thermal oxidation aging test of HTV samples was carried out for 80 days. Terahertz test results show that in the low-frequency range (0–0.2 THz), the dielectric real part of HTV first rises, then falls, and then rises until the end of aging, and the dielectric imaginary part increases with the aging time.

In the aging process of HTV, the main change is the microstructure, in which the structure of PDMS changes obviously. The FTIR results show that during the aging process, the terminal methyl group of PDMS molecules falls off, and more -OH and =O are generated. At the same time, the crosslinking between PDMS molecules leads to the denser structure and increased hardness of HTV.

The peak of $\varepsilon^{\prime \prime }$ appeared at 0.17 THz in the early aging period of HTV, which indicated that the oxidation products were formed earlier than the detectable threshold of FTIR. In the middle aging period, the main chain of PDMS breaks, which leads to the sharp decline in $\varepsilon^{\prime }$ and the decrease in polarizability. At the end of aging, the ratio of $\varepsilon^{\prime \prime }/\varepsilon^{\prime }$ increased sharply, and the loss leading mechanism warned of insulation failure.

In this paper, a nondestructive characterization method based on terahertz time–domain spectroscopy is proposed for HTV aging. The aging degree of HTV is quantitatively characterized by the ratio of dielectric peaks of real part 0.16 THz and imaginary part 0.17 THz, and the characterization of HTV aging in the early, middle, and late stages is realized.

At present, this study only analyzes the HTV samples made in the laboratory and lacks the corresponding actual HTV samples. In the future, the insulators in actual operation will be tested to verify the effectiveness of this study.

## Figures and Tables

**Table 1:** Information on related materials.

Name	CAS Numbers	Supplier	Specifications	Purity
SYLGARD™ 184 Silicone Elastomer Kit	/	Dow Corning Co., Ltd. (Midland, MI, USA)	Weight-average (M_v_) 25,000	/
Aluminum Hydroxide (ATH)	21645-51-2	Shanghai Macklin Biochemical Technology Co., Ltd. (Shanghai, China)	5000 mesh	AR
Silicon Dioxide (SiO_2_)	14464-46-1	Shanghai Macklin Biochemical Technology Co., Ltd. (Shanghai, China)	12,500 mesh	AR

**Table 2:** Relationship between infrared absorption peak of HTV and terahertz dielectric spectrum.

No.	Pearson Correlation Coefficient	*p* Value	R^2^
1	−0.3797	0.279211	0.1441
2	0.8748	0.000922	0.7653
3	0.9763	0.000001	0.9533

**Table 3:** Double Debye fitting results for HTV.

**HTV**	$\varepsilon_{\infty }$	$\Delta \varepsilon_{\mathrm{fast}}$	$\tau_{\mathrm{fast}}$	$\Delta \varepsilon_{\mathrm{slow}}$	$\tau_{\mathrm{slow}}$
	2.7709	0.2289	5.31 × 10^−13^	0.1326	6.01 × 10^−12^

## Data Availability

The original contributions presented in this study are included in the article. Further inquiries can be directed to the corresponding author.
